# Preliminary evidence-based recommendations for return to learn: a novel pilot study tracking concussed college students

**DOI:** 10.2217/cnc-2019-0004

**Published:** 2019-09-20

**Authors:** Zachary W Bevilacqua, Mary E Kerby, David Fletcher, Zhongxue Chen, Becca Merritt, Megan E Huibregtse, Keisuke Kawata

**Affiliations:** 1 Department of Kinesiology, School of Public Health, Indiana University, Bloomington, IN 47405, USA; 2 Western University College of Osteopathic Medicine of the Pacific-Northwest, Lebanon, OR 97355, USA; 3 Indiana University Health Center, Indiana University, Bloomington, IN 47405, USA; 4 Department of Epidemiology & Biostatistics, School of Public Health, Indiana University, Bloomington, IN 47405, USA; 5 Program in Neuroscience, College of Arts & Sciences, Indiana University, Bloomington, IN 47405, USA

**Keywords:** concussion, longitudinal, mTBI, phone call, recommendations, return to classroom, return to school, return-to-learn, RTL, text message

## Abstract

**Aim:**

Students re-entering the academic setting after a concussion is commonly referred to as return-to-learn and, to date, very few studies have examined the return-to-learn aspect of concussion recovery.

**Methodology:**

Nine college-aged, full-time students who were diagnosed with concussions were monitored throughout their concussion recovery. The severity for five chief symptoms (headache, dizziness, difficulty concentrating, fatigue, anxiety) were recorded six-times per day through text messages, and daily phone calls recorded participant's behavioral traits.

**Results:**

We identified five behavioral variables which significantly influenced symptom resolution (music, sleep, physical activity, water and time) (p = 0.0004 to p = 0.036). Additionally, subjects reported math and computer-oriented courses as the most difficult (33 and 44%, respectively).

**Conclusion:**

We introduce a novel approach to monitor concussed students throughout their recovery, as well as factors that may influence concussion recovery process.

## Lay abstract

Concussed students and student-athletes returning to the classroom is commonly referred as return-to-learn. To date, there are very few recommendations that can aid students’ concussion recovery in relation to their academic responsibilities. This is trouble giving that every student-athlete is first and foremost a ‘student’. In light of this, we sought to track behavioral factors that might influence the resolution of college students’ concussion symptoms as they recover from their injury and return to their coursework. Students also gave a perception of their recovery, including what types of activities and school accommodations appeared beneficial. In turn, behaviors such as minimizing music, getting more sleep, introducing more physical activity, drinking more water and overall time since injury appeared to help resolving the concussion symptoms. Additionally, students reported that math and computer-oriented courses were the most difficult, and that additional time on assignments/exams and reducing screen brightness were the most beneficial accommodations. These results are the first of their kind and will optimistically lead to recommendations utilized by healthcare professionals.

In 2012, The Atlantic listed concussion as one of the top ten ideas that changed the world [1]. The spotlight continued with the New York Times’ print of *‘110 NFL Brains*’, highlighting Dr Anne McKee and her team's work on deceased American football players [2]. As the magnifying glass grows on concussion and our focus sharpens, it is becoming ever more important to holistically examine concussion, rather than solely focusing on treating brain injury in sport. Emerging evidence has given way to protocols to ensure the safe return of athletes to sport settings [3]; however, every concussed athlete at university is first and foremost a student-athlete, with ‘student’ holding the emphasis, which supports the need for better post-concussion management in academic settings. Furthermore, it is important to be inclusive of nonathlete patients who have been previously excluded from return-to-learn (RTL) literature [4–15].

A student re-entering the academic setting after a concussion is commonly referred to as RTL. To date, very few studies have examined the RTL aspect of concussion recovery and the effects that premature classroom attendance may cause. For instance, Ransom *et al*. demonstrated that concussions can negatively impact one's perception toward academic tasks (e.g., difficulty in taking notes, studying and understanding class materials). Difficulties in perception toward linguistic and math/science classes were age-dependent, with a larger portion of high school students expressing their concern in these classes compared with their middle school and elementary school counterparts [16]. In addition, as many as 45% of concussed students may be re-entering the classroom prematurely, as defined by a recurrence or exacerbation of symptoms following after returning to the classroom [17]. Only a handful of opinion-based recommendations have been given to alleviate the worsening of concussion symptoms, such as educating academic personnel about concussion recovery and prompt initiation of academic adjustments [18]. While these preliminary studies have provided initial evidence, these data are derivative of cross-sectional studies and retrospective chart review findings.

Until now, little has been done to investigate the factors influencing safe return to academics, as evident by the scarcity of consensual evidence-based recommendations [19]. Consequently, only nine US states (IL, MA, ME, MD, NE, NY, OK, VA, VT) require RTL implementation [20]. Review of RTL literature indicates several gaps in knowledge, with disagreement among RTL protocols ranking as a chief concern [19]. The state of RTL is relatively stagnant perhaps due to the absence of sound methodology that can provide holistic evidence-based results. Therefore, we have developed a novel method for comprehensively monitoring concussed students throughout their recovery and full return to academic participation. There are two aims in this paper. First, we longitudinally investigated the potential factors influencing resolution of chief concussion symptoms in college students who were diagnosed with concussions. Second, we assessed participants’ perception of their concussion recovery and identified what types of activities and accommodations appeared beneficial.

## Methods

### Participants

Nine young adults volunteered to participate in this study. The study consisted of four male and five females with an average age of 20.2 ± 1.6 years. Inclusion criteria included being between 18 and 26 years of age, being enrolled at the studied University full time (undergraduate: 12 credit hours, graduate: 6 credit hours), possessing a cellular phone with text message and phone call capabilities and having received a diagnosis of concussion within 7 days of injury by the same sports medicine trained physician at the studied University Health Center, who was blinded from any data related to this study. Exclusion criteria included any head, neck or face injury 1 year prior to the study (e.g., concussion, eye injury); history of vestibular, ocular or vision dysfunctions (e.g., macular degeneration); pregnancy; any neurological disorders and any sleep disorders (e.g., sleep apnea, insomnia). All subjects gave written informed consent, and the studied University institutional review board approved the study.

### Study procedure

**Figure 1.**
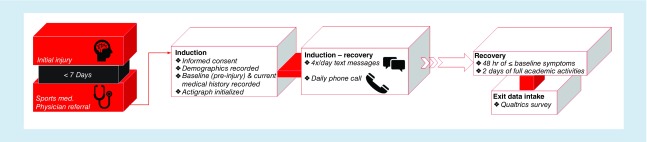
Study procedure timeline.

Upon referral from the sports medicine physician, participants met with a member of the research team to review their eligibility. Each participant was fitted with an ActiGraph wrist watch (ActiGraph wGT3X-BT) and was instructed to wear the watch during the entirety of their participation in the study, except when bathing [21]. Participants were asked to complete a Qualtrics survey (Qualtrics International Inc., Provo, UT, USA) examining their demographic information as well as previous and current medical and academic history. Some of the information gathered from this initial survey pertains to the participant's pre-injury levels (e.g., symptom scores). These data were used as the participants’ recovery criteria (see ‘Recovery criteria’ section). In an effort to limit participant's exposure to screens, the questions of this survey were asked verbally by the researcher. Next, the researcher performed a test-run to ensure that each participant's cellular phone was able to receive and respond to both a text message and phone call. Once confirmed, participants were told that they would begin receiving text messages on the same day, and their first daily phone call should be expected the following day. Refer to Figure 1 for procedural timeline.

### Activity & sleep measures

ActiGraph data were analyzed using ActiLife (version 6.5.3) software, and we employed the Sadeh algorithm to score activity level via step count and sleep duration. Sleep duration detected by the ActiGraph has been validated with 91.4–96.5% minute-by-minute agreement rates in adults compared with traditional polysomnography [21,22]. Data were scored in 60-s epochs as ‘wake’ or ‘sleep’ by measuring movement using triaxial accelerometer during each epoch, as well as movement immediately prior to and after each epoch. Subjective sleep log was used to visually inspect the ActiGraph data. Our primary interest of the total 24-h sleep (night sleep + naps) from each day until recovery was used for the statistical analysis. Similarly, we extrapolated step counts in 60-s epochs to assess activity levels, and the total step counts from each day until recovery was used for the analysis.

### Text message responses

These messages were delivered to participants at four-timepoints (9 AM, 1 PM, 5 PM, 9 PM) each day. The text messages were used to collect symptom severity of five different symptoms on a 0–10 scale, with 0 representing absence of the symptom and 10 representing the most severe level of the symptom. The text messages read as follows: “*Please rate your current symptoms. Headache, Dizziness, Difficulty Concentrating, Fatigue, Anxiety*”. If a participant did not answer the text message promptly (e.g., they were asleep, or in class), they were instructed to respond as soon as possible. These five particular symptoms were chosen based on their high prevalence postconcussion (headache, dizziness, difficulty concentrating, fatigue), [23,24] or among the population (anxiety) [25]. These symptoms captured the three symptom categories of concussion; physical, cognitive and emotional [23,24].

### Phone call responses

Phone calls were made to participants daily, at the same time (6 PM) by the same member of the research team. These calls were used to collect data regarding participant's diet (e.g., type of nutrient intake, number of meals, water/caffeine/alcohol consumption), duration of screen-time usage, music listened to, physical activity participation, medication/substance (NSAID or marijuana) usage, types of classes attended and alteration in symptom severity during the attended classes. For full list of questions, please refer to Supplementary Material 1. The current manuscript focuses our preliminary data on an association between selected behavioral factors and symptomatology; hence, all dietary factors or medication associations are not included in this manuscript. If a participant did not answer the daily phone call, they were instructed to call the researcher back as soon as possible. In case of nonrespondence, a second attempt to reach the participant was made at around 9 PM. The same researcher made each phone call based on a script. Phone call data were used in conjunction with text message data to determine recovery status, described in detail below.

### Recovery criteria

The present study utilizes diagnosis of concussion from an independent sports medicine physician as inclusion criteria; however, we did not use medical clearance from this physician as our recovery criteria. By using text message symptom data, in addition to phone call class attendance data, we were able to satisfactorily determine ‘full recovery’. Specifically, participants were required to meet the following criteria to be deemed recovered, maintain symptom levels at-or-below their individual baseline for a 48-h period (eight consecutive text messages), and attend their regular class schedule for a 2-day period without an increase in symptomatology beyond baseline. The rationale for this recovery criteria is as follows: first, not all students in attendance at the studied university carry equal levels of health insurance coverage, therefore the financial burden due to follow-up physician visits may discourage some students from returning. Second, a recent systematic review showed that concussion literature varied in their definitions of what constituted ‘full recovery’. Across the 43 included articles, 100% used a return to baseline somatic symptoms as recovery criterion, 86% used return to baseline neurocognitive testing scores, 49% used a return to physical exertion with no symptom exacerbation, only 30% included normalization of balance [26], indicating that symptom resolution is the most consistent criteria for full recovery among concussion literature. Third, a systematic review by Purcell *et al*. [9] indicates that several studies stress the need for effective communication between educators and practitioners. Therefore, it was unlikely the diagnosing physician would receive feedback from the participants’ instructors as to their academic performance or attendance. In turn, we sought to establish a conservative set of criteria that would provide us with data indicating that students had achieved a symptom-free state, in addition to having returned to their course load without symptom exacerbation.

### Exit (post-recovery) data intake

Once a status of full recovery was deemed, a member of the research team met with participants for an exit interview. This interview consisted of collecting the ActiGraph wristwatch and completion of an exit survey. This exit survey asked participants about their perceptions and experiences during their time enrolled in the study. For full list of exit interview questions, please refer to Supplementary Material 2.

### Statistical analysis

Descriptive statistics were used to analyze the demographic information and methodological efficacy, as measured by the Qualtrics survey and phone call/text message response rates, respectively. To explore the factors influencing five symptoms, we conducted a mixed-effects regression models (MRM). The model included five symptoms as outcome variables with eight independent variables; time, step count, sleep, water intake, caffeine intake, screen time, music and physical activity. The MRM was used to accommodate both repeated measurements within each day, as well as between days throughout recovery. Independent variables are expressed in different units as explained in Table 2. A positive estimate value indicates a worsening or increase in the symptom, whereas a negative estimate value indicates a resolving effect on the symptom. All MRM analyses were conducted using SAS statistical software and the level of significance was set at p < 0.05. Data for the exit survey were descriptively presented as percentages of participants that selected a given answer.

**Table 2.** Behavioral variables – symptom severity associations.

Behavioral variables	Headache		Dizziness		Difficulty concentrating		Fatigue		Anxiety	
	Estimate (SE)	p-value	Estimate (SE)	p-value	Estimate (SE)	p-value	Estimate (SE)	p-value	Estimate (SE)	p-value
Time: hour postinjury	-0.00332 (0.00121)	0.0064^†^	-0.00173 (0.00072)	0.0174^†^	-0.00295 (0.00159)	0.0647	-0.00409 (0.00135)	0.0026^†^	-0.00412 (0.00141)	0.0038^†^
Step count	0.00005 (0.00004)	0.2177	-0.00004 (0.00002)	0.0646	0.00005 (0.00005)	0.2448	0.00005 (0.00004)	0.2923	0.00006 (0.00004)	0.1336
Sleep (min)	0.00013 (0.00033)	0.7023	-0.00064 (0.00018)	0.0004^‡^	-0.0002 (0.00038)	0.5995	-0.00032 (0.00038)	0.3911	-9.11E-6 (0.00033)	0.9781
Water intake (8 oz)	0.04689 (0.04407)	0.2881	-0.04188 (0.02573)	0.1045	0.06481 (0.05607)	0.2485	0.01833 (0.04944)	0.7111	-0.1719 (0.04939)	0.0006^‡^
Caffeine intake (oz)	-0.00644 (0.02438)	0.7917	-0.01031 (0.01365)	0.4507	-0.01214 (0.02932)	0.6790	-0.03136 (0.02751)	0.2552	-0.04795 (0.02560)	0.0619
Screen time (min)	-0.00279 (0.00170)	0.1025	0.00004 (0.00095)	0.9644	-0.00368 (0.00205)	0.0730	-0.00082 (0.00192)	0.6716	0.00022 (0.00179)	0.9014
Music (min)	0.00914 (0.00264)	0.0006^‡^	-0.00064 (0.00152)	0.6714	0.00806 (0.00329)	0.0149^†^	0.007180 (0.00297)	0.0160^†^	0.00535 (0.00289)	0.0650
Physical activity (absence of)	0.6621 (0.3236)	0.0867	0.2468 (0.1798)	0.2189	1.0347 (0.3852)	0.0362^†^	1.5307 (0.3658)	0.0058^†^	0.4302 (0.3360)	0.2477

† *p < 0.05.

‡ **p < 0.001.

Data are displayed as estimate values with accompanying SEs. Level of significance set at p < 0.05.

SE: Standard error of mean.

## Results

### Demographic data & methodological efficacy

**Figure 2.**
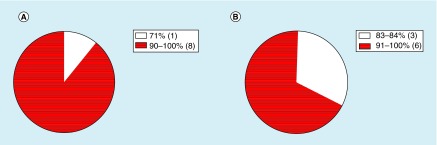
Study procedural compliance. Response rates for phone calls **(A)** and text messages **(B)**. Individual response rates are represented as part of a whole, separated by percentage ranges with accompanying (n) number of participants within that range.

The sample cohort contained nine full-time college students (five female, four male) with a mean age of 20.2 ± 1.6 years, who were diagnosed with a concussion. Demographic characteristics were described in Table 1. For responding to text messages and phone calls, the average response rates for text messages and phone calls were 92 and 93%, respectively. Further segregation of these data can be seen in Figure 2.

**Table 1.** Demographics.

Gender	5 female: 4 male
Age	20.2 ± 1.6 years
Body mass index	24.8 ± 3.0 k/m^2^
Hx of physician treatment for	
Migraines	No (89%): yes (11%)
Epilepsy or seizures	No (100%)
Substance or alcohol abuse	No (100%)
Psychiatric conditions	No (56%): yes (44%)
Diagnosed with ADD/ADHD	No (67%): yes (33%)
Diagnosed with HIV	No (100%)
Number of previous concussions	0.6 ± 1.0
Time of current injury	Morning = 3; afternoon = 5; evening = 1
Location of impact	Frontal = 5; parietal = 2; temporal = 3; occipital = 3
Cause of injury	Sport = 2; motor vehicle accident = 3; fight = 2; fall = 3
Days till recovered	18.3 ± 7.7 (Min: 10 to Max: 36)
Pre-injury symptom levels	
Headache	0.9 ± 1.5
Fatigue	1.6 ± 1.3
Dizziness	0
Difficulty concentrating	1.2 ± 1.9
Anxiety	2.3 ± 2.6
Total pre-injury symptom score	6 ± 4.7
Pre-injury behaviors:	
Hours of sleep per night	7 ± 4.8
Minutes listening to music	143 ± 92
Minutes of screen time	373 ± 182
Caffeine intake	Always = 1; often = 2; sometimes = 3; rarely = 2; never = 1
Water intake (8 oz servings)	8.8 ± 5.1
Semesters completed in a bachelors degree	3.8 ± 2.9
Number of credits between 8 and 12 PM	7.3 ± 4.3
Number of credits between 12 and 4 PM	5 ± 2.1
Number of credits between 4 and 8 PM	2.9 ± 3.3

Values are expressed as means ± standard deviation.

ADD: Attention deficit disorder; ADHD: Attention deficit/hyperactivity disorder; HIV: Human immunodeficiency virus; Hx: history.

### Beneficial factors for concussion symptom recovery

**Figure 3.**
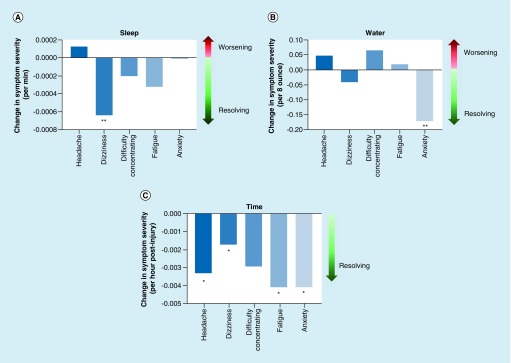
Beneficial factors for concussion recovery. Behavioral variables vs symptom severity. Music vs symptoms **(A)**, and physical activity vs symptoms **(B)** are shown respectively. Our five text message symptoms are listed along the x-axis, with symptom severity change along the left-sided y-axis, and effect of the behavior listed along the right-sided y-axis. Significance was set at *p < 0.05. *p < 0.05; **p < 0.001.

The current study identified longer sleep duration, greater water intake and overall time since injury to be beneficial factors on concussion symptom recovery (Figure 3). Sleep duration was a significant factor altering dizziness with an estimate value of -0.0006 (standard error of mean [SE] = 0.0002, p = 0.0004), indicating a 0.06% reduction in symptom severity per minute of sleep acquired over the course of recovery. Water intake was a significant factor in reducing anxiety with an estimated value of -0.17 (SE = 0.05; p = 0.006), indicating that each 8 oz serving of water consumed over the course of recovery reduced anxiety severity by 17%. Time (per hour) was a beneficial factor in ameliorating headache, dizziness, fatigue and anxiety symptoms with estimate values of -0.0033 (SE = 0.0012; p = 0.0064), -0.0017 (SE = 0.0007; p = 0.0174), -0.004 (SE = 0.0014; p = 0.0026) and -0.0041 (SE = 0.0014; p = 0.0038), respectively. Please see Table 2 for a summary of the results.

### Adverse factors for concussion symptom recovery

**Figure 4.**
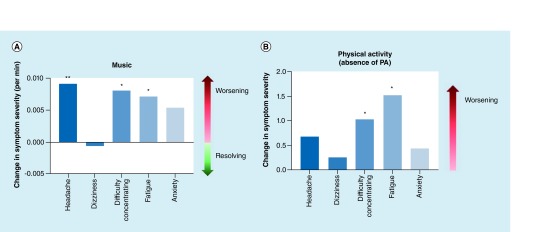
Adverse factors for concussion recovery. Behavioral variables vs symptom severity. Sleep vs symptoms **(A)**, water vs symptoms **(B)**, and time vs symptoms **(C)** are shown, respectively. Our five text message symptoms are listed along the x-axis, with symptom severity change along the left-sided y-axis, and effect of the behavior listed along the right-sided y-axis. *p < 0.05; ** p < 0.001.

Longer duration of music and absence of physical activity were shown to exacerbate symptoms (Figure 4). Time spent listening to music produced a milder increase in headache, difficulty concentrating and fatigue, resulting in estimate values of 0.0091 (SE = 0.0026; p = 0.0006), 0.0081 (SE = 0.0033; p = 0.014) and 0.0072 (SE = 0.0030; p = 0.016), respectively. These results indicate that music may increase these symptoms by 0.7 to 0.9% per minute of music listened to. Physical activity was analyzed in a binary fashion (participated or did not participate). Our results show that the absence of physical activity can significantly increase symptoms of difficulty in concentrating and fatigue, resulting in estimate values of 1.035 (SE = 0.3852; p = 0.036) and 1.53 (SE = 0.3658; p = 0.006), respectively, indicating that the absence of physical activity can exacerbate these symptoms by as much as 153%.

### Exit survey- student perspectives on recovery

Noteworthy findings, which were defined as containing at least a third of the sample size, were seen with exit interview questions 1, 2 and 4. In response to the question, “*During your recovery, which subject(s) was/were the most difficult?*”, 33 and 44% of participants expressed concerns with math and computer use during their recovery, respectively. In response to the question “*What modifications were helpful to you during your recovery?*”, 66, 56 and 33% of participants expressed that additional time on assignments/exams, reducing screen brightness and wearing sunglasses in class were helpful during recovery, respectively. Additional time on assignments/exams was selected by six participants of which 4/6 were enrolled in math-based courses, 5/6 computer-based courses and 4/6 enrolled in both. Eighty percent of participants who selected reducing screen brightness and 100% of participants who selected wearing sunglasses were enrolled in computer-based courses. At last, in response to the question “*Overall, what made you feel better?*”, participants found rest/sleep (66%) and taking breaks from screens, class and homework (44%) to be beneficial.

## Discussion

The current study identified five variables that produced a significant association with participants’ concussion symptoms. Since this study is the first to longitudinally track students’ recovery in the context of an academic setting and included a small sample size, we feel that it is premature to draw a causal inference on relationships between the observed behavioral factors and concussion symptom recovery. Therefore, in this section, we will introduce the potential mechanisms with which these factors are connected. To begin, long duration of music exposure was shown to have an adverse effect on headache, difficulty concentrating and fatigue. This observation is potentially the result of an over stimulation of cortical tissue with a concurrent reduction in cerebral blood flow supplying metabolic demands, which is supported by the previous studies that showed diminished auditory function and blood flow after a concussion [27,28]. Second, our data indicates that the absence of physical activity increased symptoms of fatigue and difficulty concentrating, corroborating the large body of emerging evidence advocating the beneficial effects of physical activity postconcussion. Literature has shown that graded physical activity induces trophic outcomes [29–34], such that when the activity is dosed correctly and in a timely manner, it can ameliorate concussion symptoms [29,30]. Therefore, it is plausible that our participants’ decision to forgo these advantageous activities may have produced rises in symptom severity. Third, longer overall sleep duration was shown to reduce the severity of dizziness among participants. The health benefits of sleep are widely understood, yet a large percentage of college-aged individuals report being sleep deprived [35,36]. Additionally, sleep deprivation has been shown to dysregulate sympathetic and parasympathetic balance of heart [37–39]. We speculate that when concussive neural damage is coupled with sleep deprivation, there is a possibility to synergistically worsen one's dizziness during the recovery phase. Forth, despite robust statistical significance, the connection between water intake and reduction in anxiety symptoms is difficult to delineate. This relationship, like others listed within this section, require further investigation. At last, time was the final variable to show significant interaction with symptoms. This association was implicit as concussion symptoms are expected to normally resolve within 10–14 days for adults and 1 month for children [34].

Exit survey reports from participants indicate two troublesome academic areas: mathematics and computer usage. The present findings corroborate previous RTL research, identifying math as the most troublesome academic subject [16]. Our results also parallel that of Ransom *et al.* [16], who reported math being the most difficult subject across multiple academic levels (freshman–junior vs elementary–high school). Functional MRI data shows that postconcussion patients exhibit a decreased blood oxygen level-dependent signal to the posterior cingulate and cerebellum when tasked with the Automated Neuropsychological Assessment Metrics Math Processing subtest, compared with healthy controls [29], indicating that concussive trauma may place a metabolic burden on brain regions that contribute to mathematical processing. Additionally, white matter tract abnormalities of the *corpus callosum* have been seen via diffusion tensor imaging in concussed pediatric patients and were in conjunction with mathematical deficits [40]. Other diffusion tensor imaging studies have also indicated mathematical performance to be associated with the commissural fibers of the *corpus callosum* [41], of which are among fibers commonly injured during concussion [42]. Data from both previous RTL literature and imaging studies corroborate our participants’ arduous perspectives toward math-based coursework, and perhaps their favorability toward additional time to complete assignments.

Reluctance toward computer usage in the present study also agrees with current literature. The concussion consensus statement most notably recommends screen time be monitored during the first stage of a graduated return-to-school protocol, steadily increasing usage throughout recovery [34]. Seemingly, this recommendation incorporates the understanding that liquid crystal display screens typically refresh 60-times per second [43]. The rapid flickering of these screens is negligible to healthy individuals, however, concussed patients with photosensitivity have find this symptom provoking [43]. This deficit may partly explain why three participants who reported higher severities of sensitivity to light during the initial postconcussion testing also selected computer usage as their most troublesome academic area and why the majority of participants enrolled in computer-based courses selected wearing sunglasses (80%) and reducing screen brightness (100%) as beneficial behaviors.

There are several strengths of the current study. To our knowledge, this is the first study to not only use text messaging to collect symptom data, but also the first study to collect symptom data as frequently as 4-times per day. The use of daily phone calls, instead of online surveys, to gather comprehensive data from participants is another innovative characteristic. There are two rationales for choosing this method: to limit exposure to electronic screens, as used by other studies [32,44], given that sensitivity to light and computer screens have been known as a chief complain of concussion [43,45], and speaking to a researcher, instead of an automated system, allows participants to ask for clarification, such as confirming that sexual activity is considered physical activity. Finally, requiring a 48-h period of academic participation, following a return to baseline symptoms, allowed us to capture participants’ tolerance to a wider range of their course load.

In contrast, the current study is not without limitations. It is important to note that symptom reports through text messages exposed participants to view a screen. There is the potential for this screen exposure to agitate symptoms, yet more than 90% of college-aged individuals use smartphones, making this a highly accessible direct line of communication between researchers and participants. Further rationale behind using text messaging was as follows: responding to symptom questions via text message takes an average of 30 s, smartphones have voice-to-text capability, allowing participants the option to vocalize their answers instead of typing and not all students have access to a personal computer for online symptom surveys. Therefore, text messaging was thought to be brief, highly effective and feasible method for repeated data collection.

The small sample size limits not only the breadth of academic majors that were included, but the various types of courses that students may be enrolled in and the level of study (undergraduate vs graduate). Additionally, a small sample size inherently limits the generalizability of the results, thus our findings are preliminary at best. Second, our criteria for recovery required a return to baseline symptomology for eight consecutive text messages, as well as 2 days of academic participation. These criteria have two limitations, the consecutive requirement of the text messages, and classes are not held on weekends which may separate the 2 days of academic participation; delaying ‘recovered’ status. Third, in an effort to minimize the time needed to answer our text messages (exposure to screens), we intentionally limited our list of symptoms to five. Unfortunately, this required us to exclude other symptoms such as sensitivity to light, sensitivity to noise and fogginess, which may have been associated with our independent variables as well. Forth, to keep our diagnosing physician unbiased in their practice, we did not track their medical recommendations or provider referrals for patients (e.g., abstinence from homework, screens, medication prescriptions, physical therapy referral, etc.), potentially influencing participant behavior.

Larger bodies of evidence-based investigations will be needed to establish a guideline that can be used in various academic settings. Until now, RTL has lacked a methodological approach to gathering such evidence-based data, therefore, we recommend researchers reproduce this study in a larger scale and at various levels of academia. A personalized concussion recovery protocol in the academic setting is the ultimate goal, but it is important to first understand the general behavioral pattern in respect to concussion recovery. Eventually, these behavior-symptom data are intended to provide practitioners with evidence-based guidance for their concussion treatments. The relationships identified in the current study are hopeful, however, we noticed that participants’ recovery trajectory and lifestyles were largely heterogeneous. Practitioners should remain cognizant of this limitation as they read this paper.

## Conclusion

The present study multimodally collected data on several variables, both independent and dependent, in a longitudinal fashion. The data provides evidence that ample sleep duration, sufficient water intake and overall time have beneficial effects on concussion symptoms during one's recovery, whereas long duration of music exposure and lack of physical activity may exacerbate concussion symptoms over time. These associations, however, must be investigated in a larger sample size if they are to be substantiated beyond early recommendations that are of profound importance to concussion care in the academic settings.

## Future perspective

Our findings introduce a novel approach to monitoring concussed students throughout their recovery. Because this evidence is the first of its kind, a large-scale exploration must be done to validate the preliminary theories. Given the robust methodology of the current study, we speculate that this will be adopted and improved upon by others, lending greater insight to establishment of RTL. It is prudent to allow the following questions to guide us: How can we make academic instructors and administrators aware of current practices for concussion management? How do these prescribed behavior modifications translate into academic accommodations? How can medical and academic personnel work together to ensure a seamless recovery for their patients/students?

**Summary points**

**Background**

- Return-to-learn (RTL) has appeared to be a secondary focus in comparison with the abundantly researched return-to-play protocols implemented ubiquitously.
- Limited studies have examined the RTL aspect of concussion recovery and the effects premature classroom attendance may cause.
- No evidence-based data has been offered regarding RTL.

**Methodology**

- Daily text message and phone calls were used to longitudinally track students, both yielding exceptional response rates.
- This is the first study to sample symptom severity data four-times per day.
- Accelerometer data were used to accurately quantify step count and sleep, versus traditional recall methods.

**Behavior-symptom associations**

- Listening to music was associated with increased headache, difficulty concentrating and fatigue.
- Absence of physical activity was associated with increased difficulty concentrating and fatigue.
- Greater water intake was associated with decreased anxiety.
- Greater amounts of sleep were associated with decreased dizziness.
- Time postinjury was associated with a decrease in all symptoms, with the exception of difficulty concentrating.

**Student perceptions**

- Math and computer-oriented courses were reported as the most difficult courses.
- Additional time on assignments/exams and reducing screen brightness were reported as the most beneficial accommodations.

**Future directions**

- The current study should be reproduced in a larger scale, and at various levels of academic settings.
- As RTL research evolves, researchers should allow the following questions to guide their exploration: When is it effective, and or safe, to implement a RTL protocol? How can student-athletes complete a RTL protocol in tandem with a return-to-play protocol? How will we include academic instructors and administrators in this process?
