# Supplementary material for: Preliminary evidence-based recommendations for return to learn: a novel pilot study tracking concussed college students
Source: Concussion. 2019 Sep 20;4(2):CNC63. doi: 10.2217/cnc-2019-0004 (PMC6787519; doi:10.2217/cnc-2019-0004)
Supplement: Supplementary file 1 [file cnc-04-63-s1.docx]

**APPENDIX A:**

Daily Phone Call Questions

“All questions are referencing the period of time between your last phone call till now”

1. “How many times have you eaten? As a reference you should count even small meals such as a handful of grapes”
2. “How many of these meals have included carbohydrates? For example, soft drinks, bread, milk, fruits, or cereals?”
3. “How many of these meals have included fats? For example, fast foods, oils, or sweets”
4. “How many of these meals have included vegetables? For example, corn, onions, or carrots?”
5. “How many of these meals and included protein? For example, fish, chicken, beans, or milk?”
6. “How many 8 oz servings of water did you drink?”
7. “How much caffeine did you drink? For example, soft drinks coffee or tea enter. As a reference a typical bottled soft drink is 16 oz?”
8. “How much alcohol did you drink? As a reference a typical can of beer is 12 oz?”
9. “What classes did you attend?”
10. ***IF THEY ATTENDED CLASSES*** “Did you felt better, worse, or no change during those classes? For example, you can say math I felt worse, art I felt no change”
11. “How much screen time have you had? For example, TV, texting, or computer”
12. “How much music have you listened to outside of class?”
13. “Have you performed any physical activity more strenuous than walking?”
14. ***IF YES*** “What was the type and duration of physical activity you performed? For example, you could say I rode a stationary bike for 30 minutes”
15. “Have you taken any medications or substances? For example, NSAIDS, marijuana, contraceptives, etc.”
