# Supplementary material for: Preliminary evidence-based recommendations for return to learn: a novel pilot study tracking concussed college students
Source: Concussion. 2019 Sep 20;4(2):CNC63. doi: 10.2217/cnc-2019-0004 (PMC6787519; doi:10.2217/cnc-2019-0004)
Supplement: Supplementary file 2 [file cnc-04-63-s2.docx]

**APPENDIX B:**

Exit Interview Questions

1. Subject Number
2. During your recovery which subject(s) was/were the most difficult?
3. What modifications were helpful to you during your recovery?
4. What type of accommodations did you find helpful? Please select all that apply.
   - Wearing sunglasses in class
   - Bringing pre-printed class notes to limit screen or PowerPoint use
   - Reducing brightness on monitors/screens
   - Recording lectures
   - Taking short breaks during class
   - Additional time to complete assignments
   - Taking tests in a quiet environment
   - Additional time to complete tests
5. Overall, what made you feel better?
6. What additional modifications and or resources do you believe may have aided in your recovery?
